# Estradiol Receptors Inhibit Long-Term Potentiation in the Dorsomedial Striatum

**DOI:** 10.1523/ENEURO.0071-23.2023

**Published:** 2023-08-02

**Authors:** Valerie J. Lewitus, Kim T. Blackwell

**Affiliations:** 1 Interdisciplinary Neuroscience PhD Program; 2 Department of Bioengineering, George Mason University, Fairfax, VA 22030

**Keywords:** dorsal striatum, estrogen, estrous cycle, long-term potentiation, sex differences, synaptic plasticity

## Abstract

Estradiol, a female sex hormone and the predominant form of estrogen, has diverse effects throughout the brain including in learning and memory. Estradiol modulates several types of learning that depend on the dorsomedial striatum (DMS), a subregion of the basal ganglia involved in goal-directed learning, cued action-selection, and motor skills. A cellular basis of learning is synaptic plasticity, and the presence of extranuclear estradiol receptors ERα, ERβ, and G-protein-coupled estrogen receptor (GPER) throughout the DMS suggests that estradiol may influence rapid cellular actions including those involved in plasticity. To test whether estradiol affects synaptic plasticity in the DMS, corticostriatal long-term potentiation (LTP) was induced using theta-burst stimulation (TBS) in *ex vivo* brain slices from intact male and female C57BL/6 mice. Extracellular field recordings showed that female mice in the diestrous stage of the estrous cycle exhibited LTP similar to male mice, while female mice in estrus did not exhibit LTP. Furthermore, antagonists of ERα or GPER rescued LTP in estrous females and agonists of ERα or GPER reduced LTP in diestrous females. In males, activating ERα but not GPER reduced LTP. These results uncover an inhibitory action of estradiol receptors on cellular learning in the DMS and suggest a cellular mechanism underlying the impairment in certain types of DMS-based learning observed in the presence of high estradiol. Because of the dorsal striatum’s role in substance use disorders, these findings may provide a mechanism underlying an estradiol-mediated progression from goal-directed to habitual drug use.

## Significance Statement

This study examined the role of the female reproductive cycle and female sex hormones in synaptic plasticity, a cellular process that underlies learning behavior, in a brain region involved in goal-directed learning. Synaptic plasticity was similar between male mice and female mice in the low estradiol phase of the cycle. In contrast, female mice in a high estradiol phase of the cycle did not exhibit synaptic plasticity. The effect of estradiol was mediated by two of the three estradiol receptors found in the brain. The results have implications for the role of the female reproductive cycle in the development of drug addiction, which involves a progression from goal-directed learning to habit-based learning as drug use switches from recreational to compulsive.

## Introduction

Estradiol, the primary form of the sex hormone class estrogen, has a number of neurologic functions, including a role in modulating learning that is associated with the dorsal striatum. This brain region is part of the basal ganglia and is involved in motor skill learning, goal-directed behavior, habit learning, and drug abuse. While sensorimotor performance is enhanced in the presence of estradiol (Becker et al., 1987), several types of learning that involve dorsal striatal circuits are impaired, such as cued learning and response learning (Zurkovsky et al., 2007; V.C. Wang et al., 2009; Neese et al., 2010).

Estradiol is also known to influence the course of addictive behavior, which heavily involves dorsal striatal circuits. Dorsal striatal activity is involved in acquisition of drug-seeking and drug-taking behaviors. During the development of substance use disorders, control over drug-taking shifts from goal-directed learning in the dorsomedial striatum (DMS) to habit-based learning in the dorsolateral striatum (DLS) as drug use switches from recreational to compulsive (Yin et al., 2009; Corbit et al., 2012; Murray et al., 2012; Gremel and Costa, 2013; Lipton et al., 2019). Higher estradiol leads to more rapid drug-taking behavior in female rats, and female rats acquire self-administration of drugs more rapidly than male rats (Lynch and Carroll, 1999; Jackson et al., 2006; Becker and Hu, 2008). In humans, observations among individuals susceptible to substance use disorders reveal that women progress into habitual drug use more quickly than men (Piazza et al., 1989; Becker, 2016; Quigley et al., 2021). The more rapid development of habitual drug-taking may be because of weaker control of reward-sensitive, goal-directed behavior by the DMS. Understanding the effects of estradiol in the DMS will further the understanding of gender differences in substance use disorder.

The three main types of estradiol receptors, ERα, ERβ, and G-protein-coupled estrogen receptor (GPER), are found throughout the dorsal striatum, but their mechanism of action in pathways underlying learning in this area is not well understood. The receptors are found exclusively outside the nucleus in dorsal striatal neurons of adult mice (Almey et al., 2012), suggesting nongenomic effects. Studies on other brain regions have found that estradiol receptors acting through intracellular signaling pathways can affect spine growth, receptor trafficking, and protein synthesis (Taxier et al., 2020). However, studies of the effect of estradiol on dorsal striatal processes are limited. ERα and ERβ are clustered with mGluR3 (G_i/o_-coupled receptor), and ERα also is clustered separately with mGluR5 (G_q_-coupled receptor; Grove-Strawser et al., 2010). The pathways activated by ER interactions with mGluR3 and mGluR5 have opposing actions on L-type calcium currents (Mermelstein et al., 1996) and cAMP response element-binding protein (CREB) phosphorylation (Grove-Strawser et al., 2010). The third ER type, GPER, leads to activation of the Gs-coupled signaling pathway (Thomas et al., 2010; Evans et al., 2016; Yu et al., 2018). It is unknown what role, if any, these ERs play in corticostriatal plasticity. While one study has found that estradiol receptor activity is necessary for long-term potentiation (LTP) in the DLS of male mice (Tozzi et al., 2015), it is unknown whether this is also the case in the DMS or in female mice. The aforementioned findings of impairment by estradiol on dorsal striatal-based learning in females suggest that the hormone may have an inhibitory effect on corticostriatal LTP.

This study tested the hypothesis that estradiol during estrus impairs plasticity in the DMS of female mice. A theta-burst stimulation (TBS) protocol was used to induce corticostriatal LTP in the DMS of C57BL/6 male and female mice. Results demonstrate that male mice and female mice in the low estradiol stage of diestrus undergo LTP, but female mice in estrus do not. Estradiol receptor agonists and antagonists were then used to determine the role of estradiol receptors in this LTP effect. It was found that blocking either ERα or GPER rescued LTP in estrous mice and activating either ERα or GPER inhibited LTP in diestrous mice. In male mice, on the other hand, ERα activation blocked LTP but GPER activation did not. The findings indicate that estradiol impairs DMS synaptic plasticity through the actions of ERα in males, and through both ERα and GPER in females.

## Materials and Methods

All animal procedures were performed in accordance with George Mason University’s institutional animal care and use committee (IACUC) regulation and followed the National Institutes of Health animal welfare standards. Every measure was taken to minimize stress and the number of mice used. C57BL/6 mice were group-housed in Teklad 7099 TEK-Fresh (wood pulp) bedding with same-sex littermates in a standard 12/12 h light/dark cycle in a light-controlled and temperature-controlled facility with *ad libitum* access to food and water.

Male and female mice aged 50–100 d were anesthetized using isoflurane and brains were extracted into oxygenated, ice-cold slicing solution (in mm: 2.8 KCl, 10 dextrose, 26.2 NaHCO_3_, 1.25 NaH_2_PO_4_, 0.5 CaCl_2_, 7 Mg_2_SO_4_, and 210 sucrose). Coronal brain slices 350 μm in thickness were created using a Leica Vibratome 1000S and were incubated in oxygenated artificial CSF (ACSF; in mm: 126 NaCl, 1.25 NaH_2_PO_4_, 2.8 KCl, 2 CaCl_2_, 1 Mg_2_SO_4_, 26.2 NaHCO_3_, and 11 dextrose) for 40 min at 33°C and then 40 min at room temperature (24–25°C). Ingredients for the slicing solution and ACSF were purchased from Fisher Scientific.

Estrous status in female mice was determined through cytological assessment of vaginal smears collected through lavage immediately following decapitation, which occurred between the hours of 12 and 2 P.M. Diestrus was characterized by a high prevalence of leukocytes and an absence or low prevalence of cornified or nucleated epithelial cells. Estrus was characterized by a high prevalence of cornified epithelial cells, an absence or low prevalence of nucleated epithelial cells, and an absence of leukocytes.

**Figure 1.**
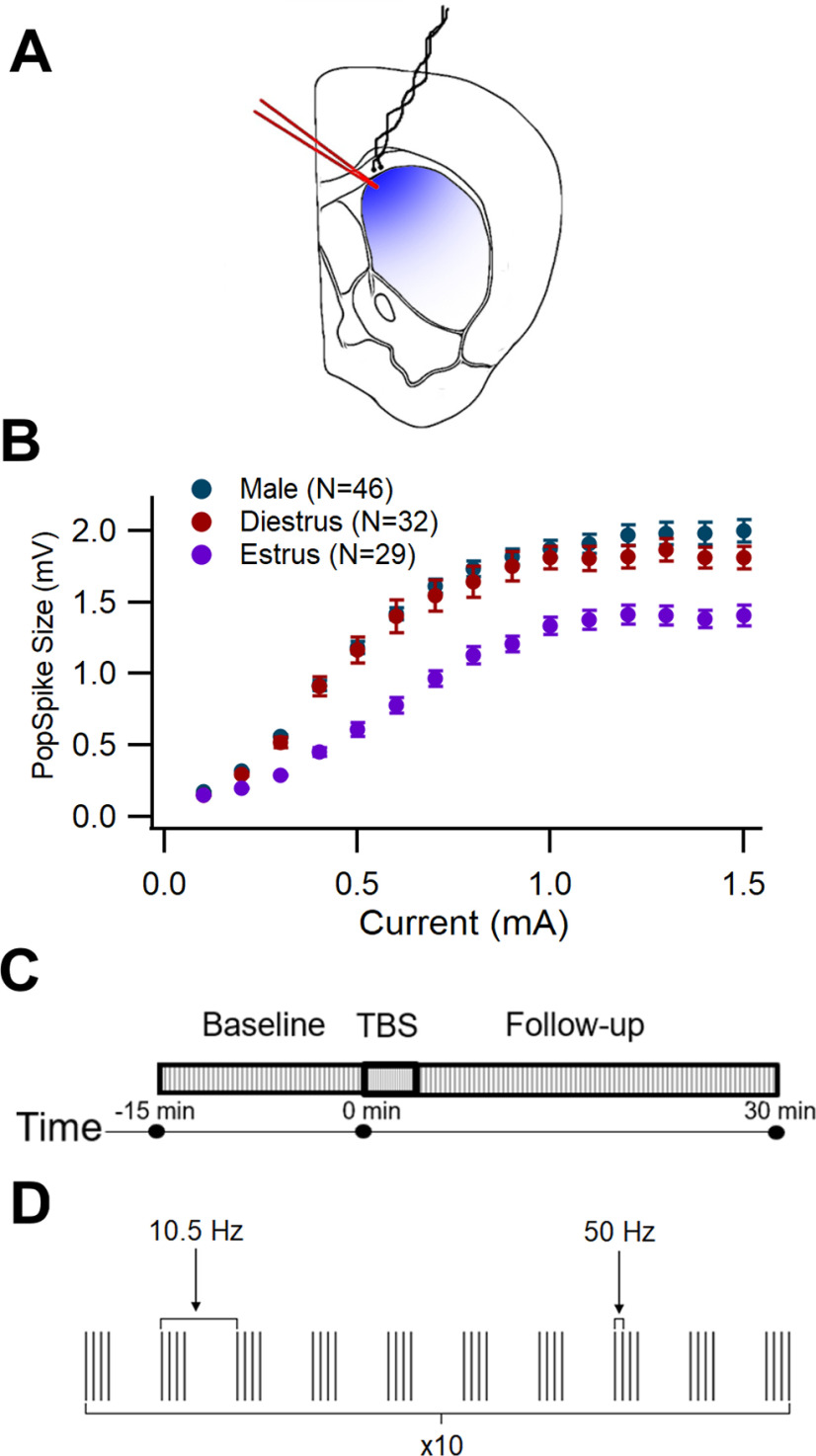
Recording protocol. ***A***, Location of stimulation electrode (black lines) in the corpus callosum and recording electrode pipette (red lines) in the dorsomedial striatum (blue area) of coronal brain slice. ***B***, Input-output curve (mean pop spike size vs stimulation current) in male, diestrous, and estrous mice. Input current ranged from 0.1 to 1.5 mA. Error bars show +/– one standard error. N: sample size. ***C***, Stimulation protocol for entire recording consisted of 15 min of baseline, theta-burst stimulation (TBS), and 30 min of follow-up. ***D***, TBS protocol consisted of 10 trains of 10 bursts at 10.5 Hz, with each burst containing four pulses at 50 Hz. One train is illustrated.

Slices were perfused with 30–32°C oxygenated ACSF containing 50 μm picrotoxin (Tocris Bioscience) at a rate of 2.5 ml/min in a submersion recording chamber. A tungsten bipolar stimulating electrode (A-M Systems, 796500) was used to inject current into the corpus callosum overlying the dorsomedial striatum (DMS; Fig. 1*A*). Current was injected in pulses of 0.05 ms. Field recordings of the voltage response from a population of neurons (pop spike) within the DMS were recorded using a borosilicate glass pipette (Sutter Instrument, BF150-75-10) containing ACSF.

Pop spike amplitude was measured by taking the absolute value of the difference between the negative peak of the trace after the stimulation artifact and either the positive peak immediately preceding the negative peak or the mean voltage of the trace immediately before the stimulation artifact, whichever was greater (Lovinger et al., 1993; Hawes et al., 2013). The signal was filtered through a four-pole, low pass Bessel filter with a cutoff frequency of 5 kHz (Warner LPF-100B), amplified by an electrometer (Warner IE-251A), and sampled at 20 kHz using LabView.

In order to determine the current amplitude to use for each experiment, an input-output curve was conducted, measuring the pop spike response across a range of current amplitudes (Fig. 1*B*). The current that would produce 40–60% of maximal response was selected for the remainder of the experiment.

The recording protocol using this selected current amplitude was comprised of three sections: a baseline recording, theta-burst stimulation, and a follow-up recording (Fig. 1*C*). The baseline pop spike recording was performed by taking measurements of pop spikes in response to a pulse every 30 s. This was run until the baseline had no discernable slope for at least 15 min. Experiments in which the baseline slope during the last 15 min exceeded ±0.01/min were excluded from analysis. Experiments with average baseline sizes exceeding 1.3 mV were also excluded.

The baseline was followed by TBS to induce long-term potentiation (LTP; Hawes et al., 2013; Fig. 1*D*). The TBS protocol consisted of 10 trains of 10 bursts with four pulses in each burst. The inter-train interval was 15 s. The interburst interval was set at a frequency of 10.5 Hz and the interpulse interval at 50 Hz. Finally, a follow-up was conducted for 30 min using the same, 0.033-Hz pulse frequency as the baseline.

During experiments with estradiol receptor agonists and antagonists, ACSF containing the drug was perfused throughout the baseline, TBS, and follow-up. Drugs were purchased from Cayman Chemical and dissolved in dimethyl sulfoxide: propylpyrazoletriol (PPT; 0.1 μm, Cayman Chemical), methyl-piperidino-pyrazole (MPP) dihydrochloride (3 μm, Tocris), 4-[2-Phenyl-5,7-bis(trifluoromethyl) pyrazolo[1,5-a]pyrimidin-3-yl]phenol (PHTPP, Cayman Chemical; 1 μm), G1 (0.1 μm, Cayman Chemical), and G15 (0.5 μm, Cayman Chemical). Concentrations were chosen based on previous usage in slice electrophysiology (Kumar et al., 2015; Bálint et al., 2016; W. Wang et al., 2018).

Data analysis was performed using custom python scripts, freely available on GitHub (https://github.com/neurord/ephys_anal/releases/tag/v1.0). Statistical analysis (Table 1) was performed using the software SAS. Figures were created through IGOR Pro, version 8.

**Table 1:** Statistical table

Manuscript reference	Data structure	Type of test	Power or confidence interval
a	No assumption	Wilcoxon two-sample test	Mean difference = −35.72 95% CI: −50.36 to −21.07
b	Normally distributed	One-factor generalized linear model	η^2^ = 0.059
b1	Normally distributed	Tukey–Kramer *post hoc* test	Mean difference = −0.098 95% CI: −0.19 to −0.0068
b2	Normally distributed	Tukey–Kramer *post hoc* test	Mean difference: 0.068 95% CI: −0.030–0.17
b3	Normally distributed	Tukey–Kramer *post hoc* test	Mean difference = −0.030 95% CI: −0.12–0.058
c	Normally distributed	One-factor generalized linear model	η^2^ = 0.34
c1	Normally distributed	Tukey–Kramer *post hoc* test	Mean difference = 0.43 95% CI: 0.12–0.75
c2	Normally distributed	Tukey–Kramer *post hoc* test	Mean difference = 0.17 95% CI: −0.12–0.47
c3	Normally distributed	Tukey–Kramer *post hoc* test	Mean difference = −0.26 95% CI: −0.53–0.0030
d	Normally distributed	One-factor generalized linear model	η^2^ = 0.074
e	Normally distributed	Analysis of covariance	Slope = −0.00095 95% CI: −0.0075–0.0056
f	Normally distributed	One-factor generalized linear model	η^2^ = 0.42
f1	Normally distributed	Dunnett’s *post hoc* test	Mean difference = 0.21 95% CI: 0.0043–0.41
f2	Normally distributed	Dunnett’s *post hoc* test	Mean difference = 0.28 95% CI: 0.064–0.49
f3	Normally distributed	Dunnett’s *post hoc* test	Mean difference = −0.042 95% CI: −0.26–0.18
g	Normally distributed	One-factor generalized linear model	η^2^ = 0.16
h	Normally distributed	One-factor generalized linear model	η^2^ = 0.35
h1	Normally distributed	Dunnett’s *post hoc* test	Mean difference = −0.37 95% CI: −0.63 to −0.10
h2	Normally distributed	Dunnett’s *post hoc* test	Mean difference = −0.32 95% CI: −0.60 to −0.053
h3	Normally distributed	Dunnett’s *post hoc* test	Mean difference = −0.39 95% CI: −0.64 to −0.13
i	Normally distributed	One-factor generalized linear model	η^2^ = 0.080
j	Normally distributed	One-factor generalized linear model	η^2^ = 0.25
j1	Normally distributed	Dunnett’s *post hoc* test	Mean difference = −0.24 95% CI: −0.44 to −0.04
j2	Normally distributed	Dunnett’s *post hoc* test	Mean difference = −0.090 95% CI: −0.26–0.080
k	Normally distributed	One-factor generalized linear model	η^2^ = 0.42
k1	Normally distributed	Tukey–Kramer *post hoc* test	Mean difference = −0.19 95% CI: −0.33 to −0.059
k2	Normally distributed	Tukey–Kramer *post hoc* test	Mean difference = 0.17 95% CI: 0.051–0.28
l	Normally distributed	Analysis of covariance	Slope = 0.12 95% CI: −0.53–0.78
m	Normally distributed	One-factor generalized linear model	η^2^ = 0.16

## Results

### Long-term potentiation does not occur in the DMS of female mice in estrus

Dorsal striatal-based behaviors and learning differ between males and females and, as seen in rodents, across stages of the estrous cycle where estradiol fluctuates (Korol and Pisani, 2015; Becker, 2016; Meitzen et al., 2018). Since LTP is a cellular mechanism of learning, this study investigated whether the observed behavioral differences may be associated with differences in LTP between sexes or across the estrous cycle.

**Figure 2.**
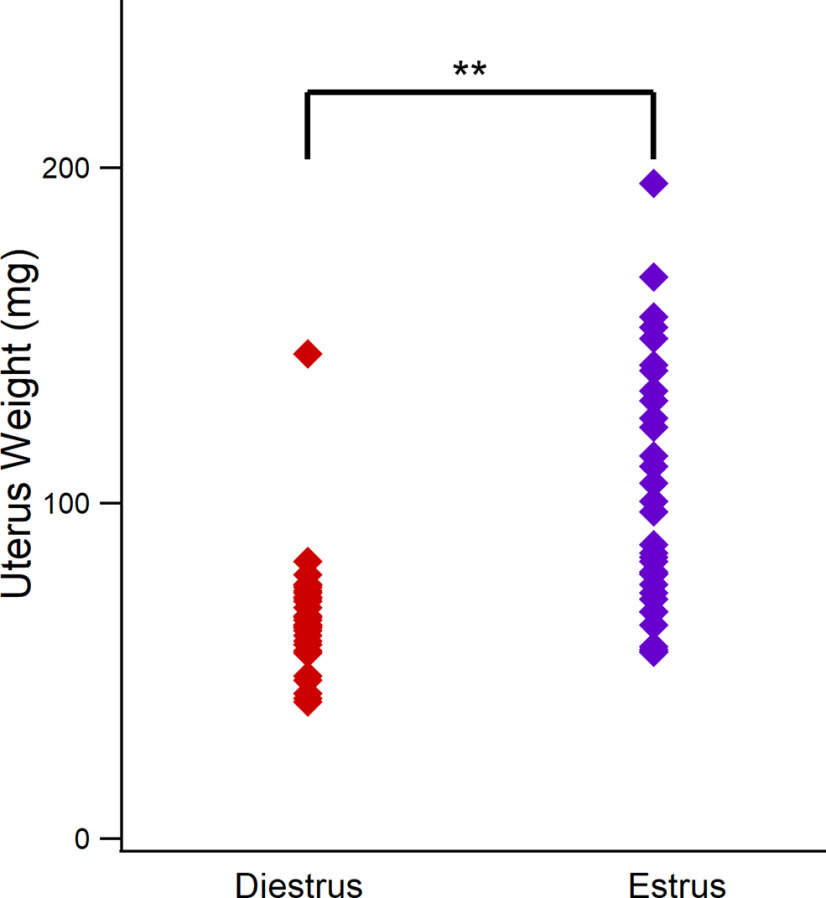
Uterus weight from diestrous and estrous mice. Each point represents a separate mouse. Mice in estrus had heavier uteri than diestrous mice (*p *<* *0.01). Each marker shows a different mouse. **: significant difference at *p* < 0.01.

LTP was induced using a TBS protocol in *ex vivo* coronal brain slices of male, female diestrous, and female estrous mice. Pop spikes before and after LTP induction were measured using extracellular field recordings. Estrous status was determined using vaginal cytology following decapitation. Uteri were extracted and weighed 15–20 min after decapitation. A Wilcoxon two-sample test determined that the mice that were categorized into the estrus stage had significantly higher uterus weights than those categorized into diestrus, Z = −4.38, *p *<* *0.0001^a^ (Fig. 2), confirming the assessment of estrous status.

Before collecting a baseline pop spike amplitude, an input-output curve was measured to select the current that produced 40–60% of maximal response. The shape of the input-output curve appeared different across sexes and estrous status: males had the greatest pop spike response to higher currents and estrous females had the lowest (Fig. 1*B*). Consequently, the mean baseline pop spike size differed among these groups: a one-factor generalized linear model showed a significant difference among males, diestrous females, and estrous females, *F*_(3,106)_ = 3.29, *p *=* *0.041^b^. A Tukey–Kramer *post hoc* test revealed the estrous group had a lower mean baseline pop spike amplitude than males (*p *=* *0.032^b1^) but not diestrous females (*p *=* *0.23^b2^). Male and diestrous female baselines did not differ (*p *=* *0.70^b3^). To ensure that the baseline size itself was not producing differences among groups, for each section below, baseline size was compared among groups, and for those with differences, the effect of baseline pop spike amplitude on LTP was tested.

**Figure 3.**
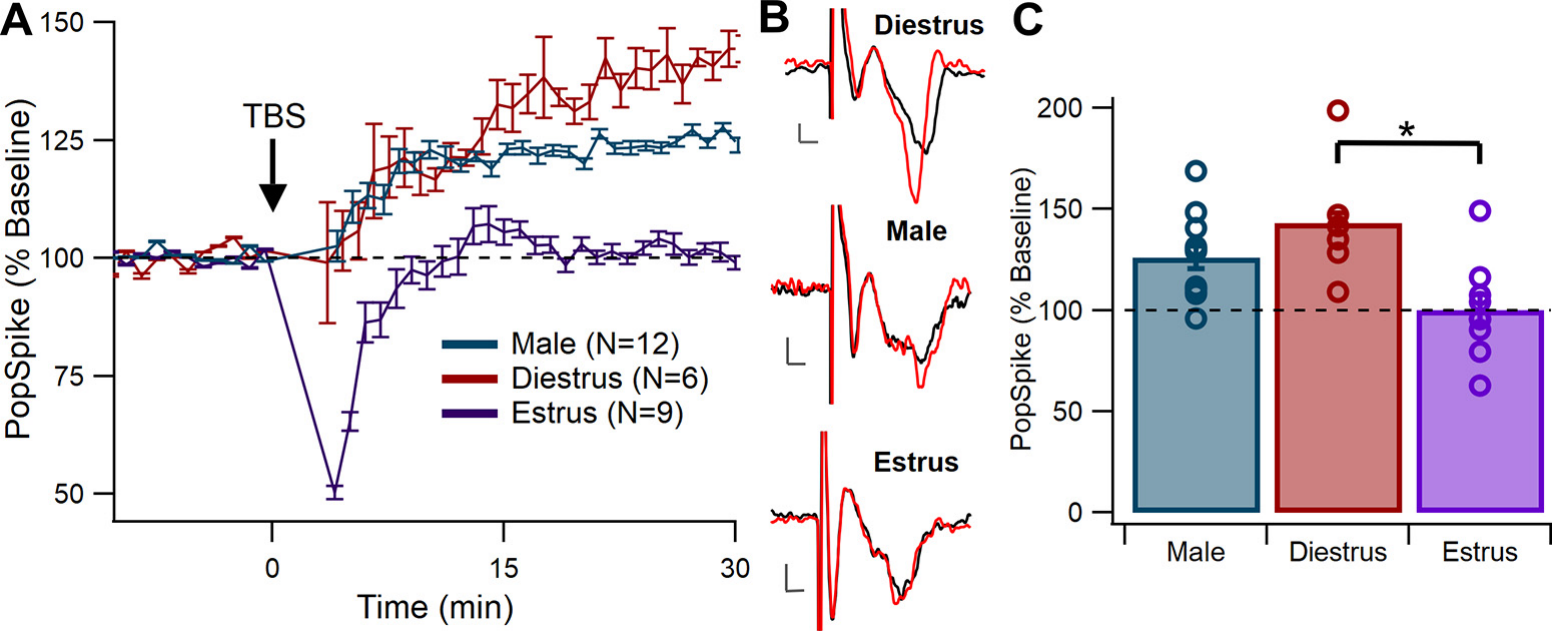
LTP occurs in males and diestrous females but not in estrous females. ***A***, Normalized pop spike size over time. Both male and diestrous females exhibited increased pop spike size after TBS (black arrow). Pop spike size in estrous females did not increase above baseline. Error bars show +/– one standard error. ***B***, Representative traces showing pop spikes before (black) and after (red) TBS. Scale bars show 0.2 mV and 1 ms. Traces were chosen based on representation of mean change at 30 min after TBS. ***C***, Comparison of normalized pop spike size at 30 min after TBS. Each marker represents a different brain slice. Diestrous females had a significantly greater normalized pop spike size compared with estrous females (*p < 0.05).

Males and diestrous females both exhibited LTP, where normalized pop spike size increased ∼25% from baseline in males and ∼40% in diestrus females, but LTP was not observed in estrous females (Fig. 3*A–C*). The estrous groups exhibited a short-term depression lasting ∼10 min after induction. A one-factor generalized linear model across male, diestrous female, and estrous female groups revealed an overall significant difference between groups at 30 min after induction (*F*_(2,24)_ = 6.29, *p *=* *0.0064^c^). Tukey–Kramer *post hoc* comparisons indicated that at 30 min, normalized pop spike size was significantly greater in diestrous females (*p *=* *0.0062^c1^) than in estrous females (Fig. 3*C*). Pop spike size at 30 min did not differ between males and diestrous females (*p *=* *0.34^c2^) or males and estrous females (*p *=* *0.053^c3^). This indicated that LTP occurred following TBS in diestrous females but not in estrous females.

To verify that estrus was the factor impairing LTP, additional tests assessed whether baseline pop spike amplitude differed between groups and whether mouse age influenced pop spike size at 30 min. A one-way generalized linear model showed no difference in baseline pop spike size between male, diestrous female, and estrous female groups in the no drug condition (*F*_(2,24)_ = 0.97, *p *=* *0.39^d^). An analysis of covariance was run using sex and estrous status as factor, and age as covariate to determine whether age predicted pop spike amplitude at 30 min. This test showed that age was not significant, (*F*_(1,23)_ = 0.09, *p *=* *0.77^e^). These results demonstrate that estrous status, and not baseline size or age, is responsible for group differences in LTP.

### ERα and GPER inhibit corticostriatal long-term potentiation in female mice

Estradiol in the striatum and other brain tissue correlates with blood plasma levels (Bixo et al., 1986); thus, it was expected that estradiol receptor (ER) activation was lower in brain slice during diestrus, a stage when estradiol levels are lowest, than during estrus. Accordingly, based on the above finding that LTP in the DMS could be induced during diestrus but not during estrus, it was hypothesized that this hormone’s actions on ERs have inhibitory effects on LTP. To test this hypothesis, TBS was run in the presence of ER antagonists and agonists to determine the ER subtypes responsible for inhibiting LTP.

**Figure 4.**
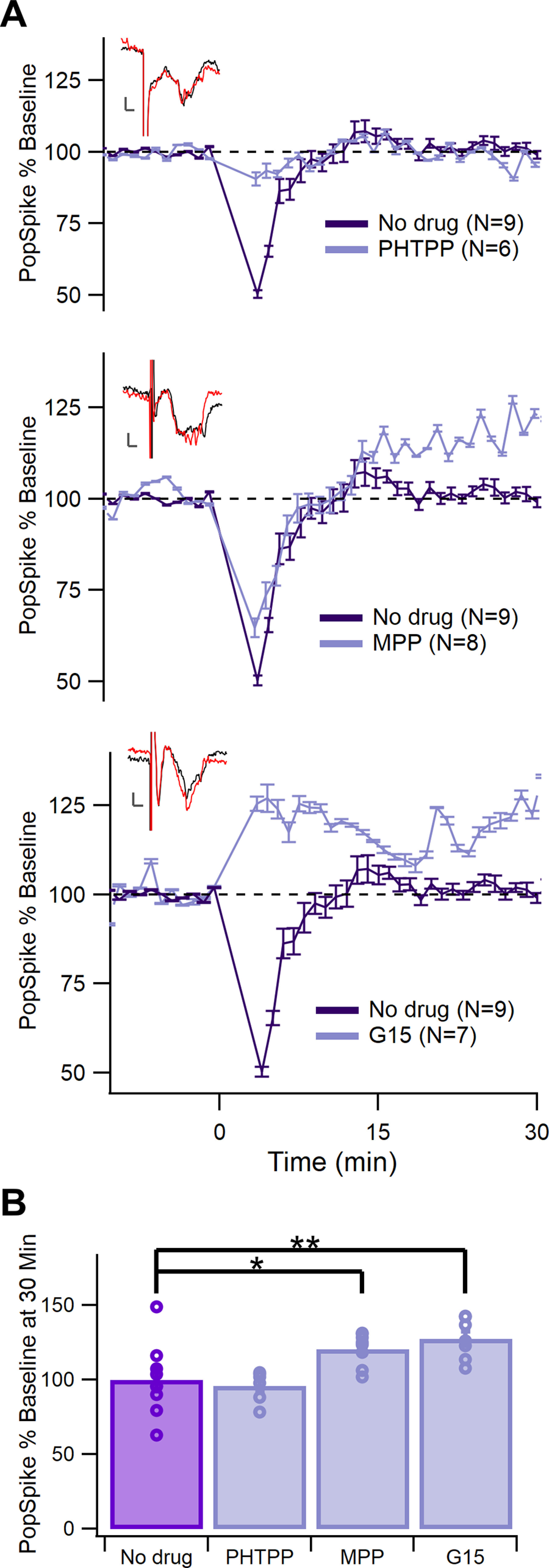
Blocking ERα or GPER rescues LTP in estrous females. ***A***, Normalized pop spike size over time in the presence (light purple) or absence (dark purple) of antagonists for ERβ (1 μm PHTPP, top), ERα (3 μm MPP dihydrochloride, middle), and GPER (0.5 μm G15, bottom). Error bars show +/– one standard error. ***B***, Comparison of normalized pop spike size at 30 min after TBS. Pop spike size increased from baseline in the presence of MPP or G15 but not in the presence of PHTPP (*p <* *0.05, **p <* *0.01).

First, this study tested whether LTP could be rescued in estrous females using antagonists of each of ERα, ERβ, or G-protein-coupled ER (GPER). The results were compared with the no drug TBS condition (Fig. 4*A*,*B*). A significant difference was found using a one-factor generalized linear model across estrous groups at 30 min (*F*_(3,26)_ = 6.15, *p *=* *0.0026^f^). A *post hoc* test using Dunnett adjustment for multiple comparisons revealed that the normalized pop spike at 30 min was significantly higher with the ERα antagonist MPP (*p *=* *0.044^f1^) and the GPER antagonist G15 (*p *=* *0.0085^f2^) than the no drug control, while the ERβ antagonist PHTPP had no effect (*p *=* *0.94^f3^). The G15 antagonist also prevented the short-term depression seen in the no drug condition. Baseline pop spike amplitude did not differ among these estrus groups (*F*_(3,26)_ = 1.71, *p *=* *0.19^g^). Thus, TBS-induced corticostriatal LTP can only be induced in the absence of either ERα or GPER activity.

**Figure 5.**
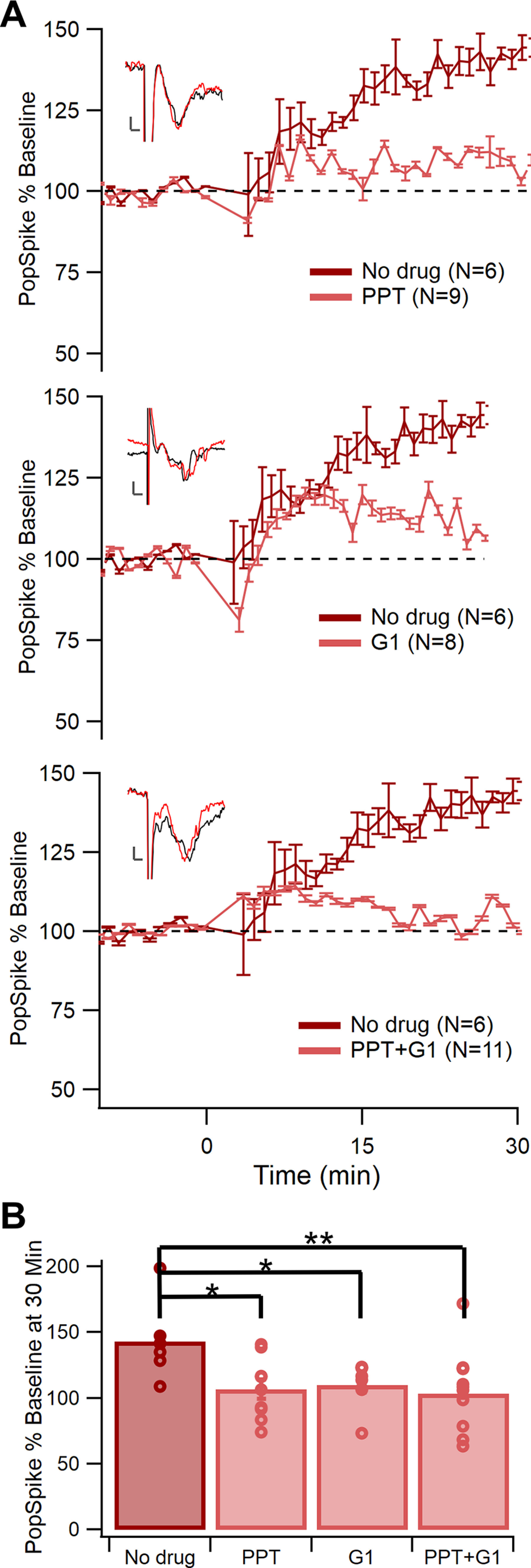
Activating ERα or GPER blocks LTP in diestrus females. ***A***, Normalized pop spike size over time in the presence (light red) or absence (dark red) of agonists for ERα (0.1 μm PPT, top), GPER (0.1 μm G1, middle), or both (bottom). Error bars show +/– one standard error. ***B***, Comparison of normalized pop spike size at 30 min after TBS. All three drug groups had a significantly lower pop spike size than the no drug group (*p <* *0.05, **p <* *0.01).

In diestrous females, TBS was run in the presence of ERα or GPER agonists to mimic a higher estradiol level and determine whether LTP would be inhibited by actions of a single ER type. Induction of LTP at 30 min was compared with the no drug diestrous group (Fig. 5*A*,*B*). ERβ agonism was excluded because of nonsignificant findings from the antagonist (Fig. 4). A main effect of drug was found using a one-factor generalized linear model (*F*_(3,29)_ = 5.27, *p *=* *0.0050^h^). A *post hoc* test using Dunnett adjustment for multiple comparisons determined that the ERα agonist PPT (*p *=* *0.0052^h1^) and the GPER agonist G1 (*p *=* *0.017^h2^) had a significantly lower pop spike magnitude than the no drug control. Because neither agonist completely blocked LTP, additional recordings were performed in the presence of the two agonists combined. LTP was further reduced in the presence of both PPT and GPER (*p *=* *0.0026^h3^). Baseline pop spike amplitude did not differ among these diestrous groups (*F*_(3,29)_ = 0.84, *p *=* *0.48^i^). Therefore, both ERα and GPER activity inhibits LTP in the dorsomedial striatum of female mice.

### ERα inhibits corticostriatal long-term potentiation in male mice

**Figure 6.**
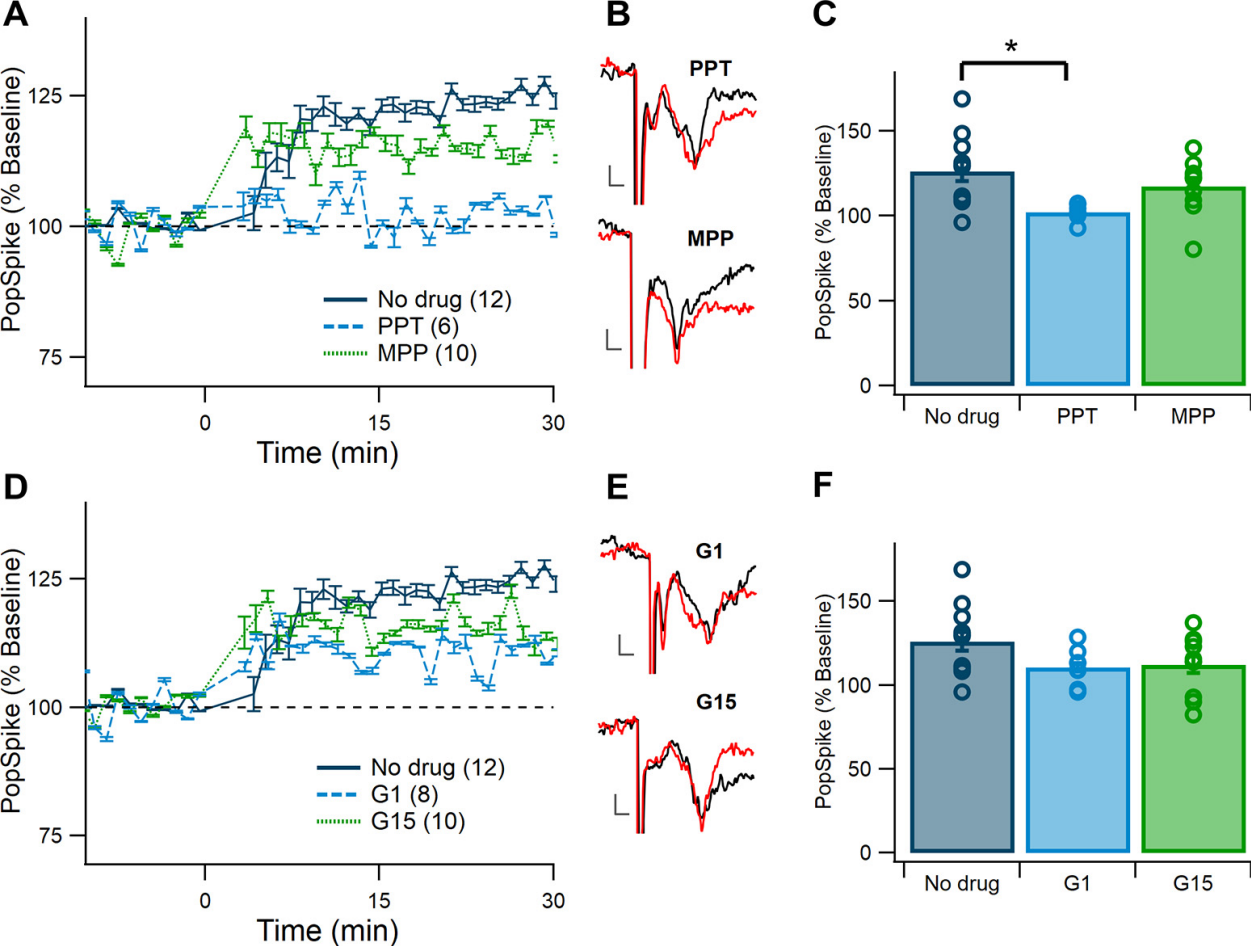
Activating ERα but not GPER blocks LTP in males. ***A***, Normalized pop spike size over time in the no drug condition (dark blue) and in the presence of the ERα agonist (0.1 μm PPT, light blue) and antagonist (3 μm MPP, green). Error bars show +/– one standard error. ***B***, Representative traces showing pop spikes before (black) and after (red) TBS with the ERα agonist (top) and antagonist (bottom). Scale bars show 0.2 mV and 1 ms. Traces were chosen based on representation of mean change at 30 min after TBS. ***C***, Comparison of normalized pop spike size at 30 min after TBS. Pop spike size in the presence of PPT was significantly lower than the no drug control (*p <* *0.05). The MPP group did not differ. ***D***, Normalized pop spike size over time in the no drug condition (dark blue) and in the presence of the GPER agonist (0.1 μm G1, light blue) and antagonist (0.5 μm G15, green). ***E***, Representative traces showing pop spikes before (black) and after (red) TBS with the GPER agonist (top) and antagonist (bottom). Scale bars show 0.2 mV and 1 ms. Traces were chosen based on representation of mean change at 30 min after TBS. ***F***, Comparison of normalized pop spike size at 30 min after TBS. No differences among groups.

The next question was whether the same effects of ERα and GPER activation and antagonism on corticostriatal LTP would be seen in male mice (Fig. 6). Males were tested because of the presence of circulating estradiol, though their levels are lower than observed in intact females (Morissette and Di Paolo, 1993; Fester et al., 2012). Additionally, males have aromatase in the dorsal striatum, indicating that there is local production of estradiol there (Küppers and Beyer, 1998). They also express similar levels of ERs as females (Küppers and Beyer, 1999; Quigley and Becker, 2021). Both agonists and antagonists were tested because, unlike in females, estradiol does not fluctuate in males, thus an increase or decrease in ER activity may shift male corticostriatal plasticity.

First, the role of ERα in LTP in males was tested. The ERα agonist PPT resulted in a clear decrease in pop spike size from the no drug condition (Fig. 6*A*), similar to what was observed in diestrous females (Fig. 5). The ERα antagonist MPP slightly reduced pop spike size (Fig. 4). To determine whether these observed differences were significant, a one-factor generalized linear model was run and a main effect of ERα drug was found (*F*_(2,25)_ = 4.06, *p *=* *0.030^j^). Dunnett’s *post hoc* comparisons indicated that the ERα agonist PPT produced a significantly lower pop spike size at 30 min than the no drug control (*p *= 0.017^j1^). The antagonist MPP did not significantly change pop spike size from the no drug control group (*p *=* *0.38^j2^; Fig. 6*C*). Baseline pop spike size differed among the male groups (*F*_(2,25)_ = 9.21, *p *=* *0.001^k^), where the no drug condition was significantly lower than the PPT (*p *=* *0.0041^k1^) and MPP (*p *=* *0.0039^k2^) groups. Despite this difference, pop spike size at 30 min was not predicted by baseline pop spike amplitude (*F*_(1,24)_ = 0.16, *p *=* *0.70^l^). This indicates that ERα activation inhibits LTP in the male mouse DMS but, as expected in low levels of estradiol, LTP is not enhanced on inhibition of ERα.

When LTP induction was tested in the presence of the GPER agonist G1 or the GPER antagonist G15, both drugs appeared to decrease pop spike size by about half (Fig. 6*D*). However, statistical analysis using a one-factor generalized linear model revealed no differences between the no drug condition and the G1 and G15 groups (*F*_(2,27)_ = 2.50, *p *=* *0.10^m^; Fig. 6*F*). These results indicate that, unlike in females, GPER activation does not prevent LTP induction in male mice.

## Discussion

This study examined the effect of the estrous cycle and estradiol receptors (ERs) on synaptic plasticity in the dorsomedial striatum (DMS). The results showed that long-term potentiation (LTP) can be induced using theta-burst stimulation (TBS) in the DMS of diestrous mice, and that the amplitude is comparable to male mice, but that LTP induction is hindered during the estrous stage of the cycle. Experiments further demonstrated that ERα and GPER are modulating LTP induction. Specifically, corticostriatal LTP occurs in estrous mice when either ERα or GPER is blocked. In diestrous mice, when circulating estradiol levels are low, using an ERα or GPER agonist impairs LTP. In male mice, the ERα agonist impairs LTP but the GPER agonist does not, and neither the ERα nor the GPER antagonist has a significant effect. Collectively, these findings demonstrate a novel role of estradiol in corticostriatal LTP and offer a new context for the current understanding of the hormone’s actions in the DMS.

Estradiol may be controlling LTP through its modulation of striatal neuron properties. Spiny projection neurons (SPNs) in the dorsal striatum of mice exhibit reduced excitability during estrus compared with other stages of the cycle, as evidenced by a higher rheobase, higher action potential threshold, and lower action potential amplitude (Willett et al., 2020). The role that ERs play in these changes is unclear. In the rat ventral striatum, miniature EPSC (mEPSC) frequency fluctuates across the estrous cycle and estradiol modifies mEPSC frequency and neuron excitability (Proaño et al., 2018, 2020; Krentzel et al., 2019). However, in the rat dorsal striatum, no effect of estradiol on excitability has been observed, regardless of estrous status (Krentzel et al., 2019). These observations in the rat striatum do not imply that estradiol does not play a role in mouse dorsal striatal SPN properties: there may be an interspecies difference in estradiol action on SPNs, or it is possible that evoked, instead of spontaneous, synaptic input is altered by estradiol. Alternatively, while SPNs are a logical target to study estradiol’s effects in the striatum because they comprise over 90% of striatal neurons (Kemp and Powell, 1971; Bolam et al., 2000), it cannot be ruled out that the effect of estrus is because of estradiol action on interneurons, although a recent related study found no evidence of estradiol affecting cholinergic interneuron excitability (Kövesdi et al., 2023). Another possibility is that estradiol’s effect on striatal circuitry underlying LTP may come from another source, such as dopamine neurotransmission.

Dopamine is critical for synaptic plasticity in the DMS; thus, it is crucial to consider estradiol’s effect on dopamine as a mechanism for controlling LTP. Higher estradiol levels result in higher dopamine availability in the dorsal striatum, as evidenced by higher basal extracellular dopamine during estrus than during diestrus (Xiao and Becker, 1994) and by increased stimulated dopamine release in the striatum during estrus or after estradiol infusions (Becker and Beer, 1986; Becker, 1990; Castner et al., 1993; Becker and Rudick, 1999; Shams et al., 2016; Calipari et al., 2017; Song et al., 2019). This increased release may be an indirect result from estradiol’s reduction of GABA release (Hu et al., 2006; Schultz et al., 2009). Despite the consensus on the effect of estradiol on dopamine release, the effect that enhanced dopamine has on plasticity is less clear. Heightened dopamine availability could have opposing effects on plasticity, depending on which downstream targets are affected. Corticostriatal LTP requires dopamine D1 receptor activity (Calabresi et al., 2000; Kerr and Wickens, 2001; Reynolds and Wickens, 2002; Hawes et al., 2013). On the other hand, D2 receptor activity induces long-term depression (LTD) through retrograde endocannabinoid signaling (Gerdeman et al., 2002; Kreitzer and Malenka, 2005). Perhaps the absence of LTP seen in estrus is because of increased dopamine binding to D2 receptors. Indeed, some studies have found that estradiol increases D2 receptor density (Fernández-Ruiz et al., 1989; Ursic et al., 2003); however, these effects are dependent on factors such as estradiol dosage and length of time applied (Roy et al., 1990; Bazzett and Becker, 1994). D2 receptors are involved in LTD through D2-receptor-containing SPNs (Calabresi et al., 1992; Kreitzer and Malenka, 2005) as well as through reduction of cholinergic interneuron activity, which can facilitate LTD in both D1R and D2R SPNs (Z. Wang et al., 2006). Future studies comparing LTP between identified D1-receptor-containing and D2-receptor-containing striatal neurons may be able to determine whether estradiol’s effect on dopamine is responsible for the impairment in LTP.

There are a variety of other mechanisms through which ERα and GPER could be affecting LTP in the dorsal striatum. From research in males, corticostriatal LTP involves depolarization of SPNs, leading to calcium influx and subsequent activation of kinase pathways that cause LTP (Kreitzer and Malenka, 2008; Lovinger, 2010). Estradiol receptors could be targeting any of the components of this pathway. One possible mechanism is through reducing calcium influx through voltage-dependent calcium channels because of a reduction in excitability (Willett et al., 2020). This lower calcium would reduce the activation of calcium-dependent kinases critical for LTP, such as calcium/calmodulin-dependent protein kinase II (CaMKII; Q. Wang et al., 2017) and protein kinase C (PKC; Gubellini et al., 2004; Hawes et al., 2013). For more specific methods through which estradiol affects LTP, pathways activated by individual receptors, ERα and GPER, must be explored.

ERα coupling to mGluR may be its mechanism of action in inhibiting LTP. A subset of ERα activates mGluR5 (Grove-Strawser et al., 2010), which is a G_q_-coupled receptor. The level of calcium influx may determine whether Gq signaling leads to LTP or LTD (Kim et al., 2013). Under this model, a higher calcium level leads to processes that result in LTP via PKC activation, and a lower calcium level leads to processes that result in LTD via production of the endocannabinoid 2-arachidonoylglycerol (2-AG). As previously discussed, the lower excitability of SPNs during estrus could result in lower calcium influx in response to cortical input. Perhaps the ERα-mediated Gq activity in a lower-calcium environment results in the occurrence of LTD in some synapses after TBS, leading to no net increase in synaptic strength as measured by field recording. Another downstream target of mGluR5 is extracellular signal-regulated kinase (ERK; Choe and Wang, 2001; Yang et al., 2004; Mao et al., 2005), whose downstream targets lead to LTP (Xie et al., 2009; Cerovic et al., 2013; Hawes et al., 2013), as well as LTD (Cui et al., 2011) and depotentiation (Cerovic et al., 2015), although the conditions that determine the end result of ERK phosphorylation are unclear. This route indicates that ERα could be preventing bidirectional plasticity, and further investigation should be conducted into whether LTD occurs in the presence of ERα activation.

Another route through which LTP could be inhibited by ERα is through the ER’s association with another type of mGluR. A separate subset of ERα activates the G_i/o_-coupled receptor mGluR3 (Grove-Strawser et al., 2010), which is located presynaptically on the terminals of cortical neurons and whose activation reduces calcium influx, and therefore glutamate release (Kupferschmidt and Lovinger, 2015). Less glutamate release would reduce the activation of postsynaptic glutamate receptors on SPNs involved in LTP induction, including AMPA and NMDA receptors, which are required for LTP (Calabresi et al., 1992; Partridge et al., 2000; Malinow and Malenka, 2002; Malenka, 2003; Lüscher and Malenka, 2012; Hawes et al., 2013). An ERα mediated increase in mGluR3 activity on cortical terminals could therefore be a separate mechanism through which ERα can affect LTP. ERα mediated changes in mGluR3 activity also may explain the reduced response to current injection seen in the input-output curve in the present study (Fig. 1*B*).

GPER’s mechanism of action in inhibiting LTP could be either through its own G-protein activity or through the reinforcement of ERα activity. GPER is a Gs-coupled receptor (Thomas et al., 2010; Filardo and Thomas, 2012; Evans et al., 2016; Yu et al., 2018). While Gs activity is known to be involved in LTP through its downstream target protein kinase A (PKA; Spencer and Murphy, 2002; Hawes et al., 2013), a negative feedback loop involving elevated activity of phosphodiesterase (PDE) consequent to prolonged elevation of Gs activity could act paradoxically to reduce PKA activity during LTP (Lim et al., 1999; Houslay and Adams, 2003). Specifically, a higher basal level of Gs pathway activity throughout proestrus and estrus because of estradiol activation of GPER could result in enhanced PKA and PDE activity during estrus, which can reduce cAMP activity during TBS, thereby inhibiting PKA pathways required for LTP. Alternatively, GPER could be enhancing the activity of ERα. The interaction between these two receptors (Micevych and Christensen, 2012; Bourque et al., 2015; Acramel and Jacquot, 2022) could explain how either GPER activation *or* ERα activation inhibits LTP in diestrous females. ERα may be allosterically modulated by GPER to prevent LTP, which would explain why GPER inhibition alone is sufficient to rescue LTP. This mechanism may also explain why GPER activation did not prevent LTP in males; perhaps the estradiol level in males is too low to produce a high enough level of ERα activity for GPER modulation to have an effect. There are thus many possibilities through which these receptors could be inhibiting LTP, and this will be an important area of research moving forward, as it is crucial to understanding the complexity of estradiol’s involvement in general synaptic dynamics underlying learning.

The LTP deficits uncovered by this study may underlie learning impairments in dorsal striatum-based behaviors observed in the presence of high estradiol. Dorsal striatal learning is underpinned by synaptic plasticity (Reynolds et al., 2001; Yin et al., 2009; Shan et al., 2014, 2015; Hawes et al., 2015). The inhibition of LTP would be expected to result in poorer performance in learning tasks. Reflecting the current study’s finding that ER activity inhibits LTP, past research has demonstrated that several types of learning that involve the dorsal striatum are impaired in the presence of higher estradiol levels. Rats given subcutaneous estradiol perform worse in cued learning (Galea et al., 2001; Daniel and Lee, 2004; V.C. Wang et al., 2008, 2009), response learning (Davis et al., 2005; Pleil et al., 2011), and spatial alternation (V.C. Wang et al., 2008, 2009; Neese et al., 2010). All of these learning strategies are known to involve dorsal striatal circuits (Packard et al., 1989; Packard and McGaugh, 1996; Balleine, 2005; Yin et al., 2005a, b; Lee et al., 2008; Moussa et al., 2011). A particularly well-characterized example of estradiol’s differential effects on learning is the switch in learning types from dorsal striatal-based learning strategies to hippocampal-based learning strategies. The bias in learning strategy between dorsal striatum-based learning and hippocampal-based learning fluctuates across the estrous cycle according to estradiol level, where higher estradiol stages favor place learning over response or cued learning (Korol et al., 2004; Yagi et al., 2017). This bias toward hippocampal strategies over dorsal striatal strategies can also be observed when estradiol is injected subcutaneously (Korol and Kolo, 2002; Davis et al., 2005; Quinlan et al., 2008; Hussain et al., 2016) or directly into the dorsal striatum (Quinlan et al., 2013). One study found that subcutaneous injection of the ERα agonist PPT alone is enough to produce this shift in learning strategy (Pisani et al., 2016). Estradiol also enhances other types of hippocampal-based learning such as object placement and recognition memory (Walf et al., 2006; Paris and Frye, 2008; Inagaki et al., 2010, 2012; Phan et al., 2012, 2015; Jacome et al., 2016; Luine et al., 2018). Consistent with this switch, estradiol is necessary for and enhances synaptic plasticity in hippocampus (S.G. Warren et al., 1995; Foy et al., 1999; Smith and McMahon, 2006; Grassi et al., 2011; Hasegawa et al., 2015; Lu et al., 2019). In addition, excitability and spine density in the hippocampus both fluctuate across the estrous cycle, with highest excitability during proestrus and estrus, and higher spine densities observed during stages with high estradiol and progesterone (Kubo et al., 1975; Woolley and McEwen, 1992; Scharfman et al., 2003; Kato et al., 2013; Jaric et al., 2019). Therefore, the results of the current study are congruous with the growing body of literature indicating that estradiol’s effect on synaptic plasticity parallels its effect on the learning types that involve this plasticity. In other words, estradiol may be a regulator of behavioral strategy through control of synaptic plasticity, e.g., selecting between hippocampal and striatal strategies depending on high versus low estradiol, respectively.

The relevance of these results to behavior may extend to pathology as well. A major component of striatal-based behavior that is influenced by estradiol is habit formation, which contributes to addictive behaviors. Among individuals in treatment for drug abuse, women are observed to develop habitual drug use more quickly than men (Piazza et al., 1989; Becker et al., 2017; Quigley et al., 2021) and can exhibit different levels of craving and relapse across the menstrual cycle (Allen et al., 2009; Franklin et al., 2015; Carlson et al., 2017; Hayaki et al., 2020; J.G. Warren et al., 2021). In rodent studies, chronic estradiol treatment leads to quicker acquisition of cocaine, nicotine, and methamphetamine self-administration than in estradiol-depleted females (Jackson et al., 2006; Larson et al., 2007; Kucerova et al., 2009; Flores et al., 2016). These effects, however, are dose and drug dependent, as increasing estradiol doses can reduce cocaine intake (Hu and Becker, 2008), and estradiol-depleted rats exhibit higher opiate self-administration than those treated with estradiol (Sharp et al., 2021). During habit formation, behavior changes from goal-directed to goal-independent (habitual) as control over drug intake switches from the DMS to the dorsolateral striatum (DLS; Corbit et al., 2012; Malvaez, 2020). It has been hypothesized that the control does not simply shift, but that there is a competition between these two subregions, where stronger connections in one subregion will bias dominance over behavior to that subregion. Indeed, inhibiting the DLS produces a return to goal-directed behavior and inhibiting the DMS produces habitual behavior (Yin and Knowlton, 2006). Perhaps higher estradiol promotes the development of habitual behavior as it weakens DMS-based, goal-directed learning. The effect of estradiol in the DLS of females is unknown, but in males it has been demonstrated to be necessary for LTP (Tozzi et al., 2015). Estradiol’s action on the mechanisms underlying learning in the dorsal striatum may be a key component in understanding the hormone’s role in substance use disorders.

## Synthesis

Reviewing Editor: Tod E Kippin, University of California Berkeley Decisions are customarily a result of the Reviewing Editor and the peer reviewers coming together and discussing their recommendations until a consensus is reached. When revisions are invited, a fact-based synthesis statement explaining their decision and outlining what is needed to prepare a revision will be listed below. The following reviewer(s) agreed to reveal their identity: Devin Mueller.

I have received two independent reviews of our manuscript which both expressed a very high level of enthusiasm for the findings of your study in general, particularly regarding the novelty of investigating the impact of sex and hormones on LTD. However, both expressed some concerns that require moderate revision and clarifications be made before a final decision can be made. As such, I am recommending the manuscript be revised and re-reviewed.

Below are specific comments from the reviewer that should be carefully addressed prior to submitting a revised manuscript.

Reviewer 1

In the present study, the authors examine the effects of estradiol and its receptors on theta-burst stimulation-induced long-term potentiation (LTP) in dorsomedial striatal neurons. Extracellular field recordings in the dorsomedial striatum (DMS) from brain slices derived from intact male and female mice were performed. Whereas theta-burst stimulation induced LTP in the DMS of male mice, LTP was only observed in the DMS of female mice in diestrus. When female mice were sacrificed during estrus, LTP was not observed. Inhibiting the estradiol receptors ERa and GPER rescued LTP in in estrus mice whereas activating ERa and GPER impaired LTP in diestrus mice. These data suggest that estradiol inhibits LTP in the DMS of females through activation of ERa and GPER receptors.

These data extend our understanding of the effects of estradiol in the DMS, suggesting that high levels of estradiol impair learning and may promote habit formation. The study is straightforward and expands our understanding of cellular mechanisms of estradiol on neuronal plasticity within the DMS. However, I have some concerns with the study in its present form.

1. Although the authors have demonstrated that estradiol inhibits LTP, the mechanisms of action are unclear. Both ERa and GPER receptors are targeted suggesting these receptors mediate the inhibition of LTP. How they do so, however, is not examined directly and limits our understanding. Furthermore, there is no clear assay to assess the relevance of these findings (such as a behavioral readout).

2. In Figure 4, GPER inhibition with G15 not only rescues LTP but does so in a manner similar to males in Figure 3 rather than like diestrus females. Does GPER activation in males impair LTP in the DMS? Examining the effects of ER agonists and antagonist in the DMS of males would be insightful.

3. Minor: In the statistical table, the first manuscript reference should be “a”.

Reviewer 2

This is an interesting study that outlines how estradiol affects synaptic plasticity in the dorsomedial striatum (DMS). The authors show that female mice in diestrus stage exhibited LTP similar to male mice; however, female mice in estrus did not exhibit LTP at all. Then using pharmacological approaches with estrogen receptor agonists and antagonists they found that ERα or GPER likely play a critical role in this process as antagonism of both receptors can rescue LTP in estrus females, and agonism can induce LTP in diestrus females. Overall this is a simple, but interesting manuscript that is important for the field. There were a few relatively minor comments that should be addressed:

How are the antagonists working in slice. Is there estradiol tone?

How were the doses chosen for the pharmacology studies? The doses are listed but no information on how they were chosen is provided.

“Estradiol, the predominant form of the sex hormone estrogen” is stated in the first sentence of the abstract - Estrogen is not a hormone, it is a class of hormones (estrone, E1; estradiol, E2; and estriol, E3). The authors should go through out and make sure that their language is appropriate throughout.

## Author Response

Reviewer #1:

These data extend our understanding of the effects of estradiol in the DMS, suggesting that high levels of estradiol impair learning and may promote habit formation. The study is straightforward and expands our understanding of cellular mechanisms of estradiol on neuronal plasticity within the DMS. However, I have some concerns with the study in its present form.

1. Although the authors have demonstrated that estradiol inhibits LTP, the mechanisms of action are unclear. Both ERa and GPER receptors are targeted suggesting these receptors mediate the inhibition of LTP. How they do so, however, is not examined directly and limits our understanding. Furthermore, there is no clear assay to assess the relevance of these findings (such as a behavioral readout).

Response: Thank you for the suggestion, as it encouraged us to think beyond the surface of what could be happening with these receptors. We have completely rewritten most of the

Discussion (lines 259, 277 to 278, and 300 to 357) to better explain the possible mechanisms through which the receptors could be inhibiting LTP. Testing which of the following mechanisms is actually responsible for our observed results is part of our future directions.

One possible mechanism is through reducing calcium influx through voltage-dependent calcium channels due to a reduction in excitability (Willett et al., 2019). This lower calcium would reduce the activation of calcium-dependent kinases critical for LTP. Alternatively, ERα coupling to mGluR may be its mechanism of action in inhibiting LTP (Grove-Strawser et al., 2010). Enhanced mGluR5 activation, together with lower calcium levels during estrus may enhance the downstream Gq signaling pathways involved in DAG conversion into the endocannabinoid 2-AG. Another scenario is that ERα acts through mGluR3 presynaptically, where its activation of Gi/o-coupled signaling reduces glutamate release into the DMS, and thus reduces the effectiveness of glutamatergic signaling that initiates LTP.

We also describe two hypotheses for GPER’s role in inhibiting LTP. The first is that LTP is inhibited (paradoxically) through the GPER Gs-coupled pathway. Higher basal levels of GPER activity during the high-estradiol periods of proestrus and estrus could result in a compensatory buildup of PDE4 activity, which could then reduce cAMP activity during the theta burst stimulation protocol. The other hypothesis is that GPER works to enhance the effect of active ERα, as the two ERs are known to co-localize in other tissues (Acramel and Jacquot, 2022) and may also do so in the dorsal striatum. The results in males - that the GPER agonist did not inhibit LTP - also are consistent with this hypothesis, under the prediction that the estradiol level in males is too low to activate enough ERα for the GPER enhancement to have an effect.

The behavioral assay is an excellent suggestion and would be an important study to confirm the relevance of our findings. It would be difficult to study the estrous cycle in a DMS-based learning task because the training protocols that measure these types of learning take multiple days - longer than the length of an estrous cycle phase. Alternatively, ovariectomized rats with or without estradiol replacement could be used to measure ER action. This would still be a monumental undertaking, due to the need to demonstrate the role of estradiol specifically in the striatum. In other words, we would need to give infusion of ER agonists and antagonists directly into the striatum for the duration of the training protocol. Unfortunately, while this might demonstrate the role of estradiol in learning, it would not necessarily demonstrate how the interaction between estradiol receptors and LTP impact behavior. In fact, demonstrating the role of LTP in behavior is exceedingly difficult (Whitlock et al., 2006). In summary, though showing the behavioral relevance of estradiol inhibition of LTP would be a huge contribution to the field, such an experiment is beyond the scope of our current research. However, we have rewritten a paragraph in our Discussion (lines 358 to 392) that now gives a better overview of past behavioral experiments that test the effect of estradiol on dorsal striatal learning in rats.

2. In Figure 4, GPER inhibition with G15 not only rescues LTP but does so in a manner similar to males in Figure 3 rather than like diestrus females. Does GPER activation in males impair LTP in the DMS? Examining the effects of ER agonists and antagonist in the DMS of males would be insightful.

Response: We have conducted additional experiments that examine the effects of ERα and GPER agonists and antagonists on LTP in male DMS (Fig. 6; Results lines 217 to 245, lines 820 to 832). The results are quite interesting, and demonstrate that ERα activation inhibits LTP in males, which is consistent with our observations in diestrus females. Consistent with the observation that estradiol levels of males are lower than females, the ERα antagonist had no effect on LTP in males. In contrast to results in diestrus females, we found that GPER does not impair LTP in males, revealing a sex difference in GPER function. We hypothesize that GPER activation is facilitating ERα activity in females, and this interaction requires a minimal basal level of estradiol, which is lacking in males (Discussion lines 351 to 354).

Additionally, we were interested in the short-term depression seen in estrus females, as it was prevented by the GPER antagonist and it occurred in diestrus females under the G1 agonist. This short-term depression did not appear in males with the GPER agonist, reinforcing the conclusion that GPER has different effects in males and females.

3. Minor: In the statistical table, the first manuscript reference should be “a”.

Response: Fixed!

Reviewer #2:

This is an interesting study that outlines how estradiol affects synaptic plasticity in the dorsomedial striatum (DMS). The authors show that female mice in diestrus stage exhibited LTP similar to male mice; however, female mice in estrus did not exhibit LTP at all. Then using pharmacological approaches with estrogen receptor agonists and antagonists they found that ERα or GPER likely play a critical role in this process as antagonism of both receptors can rescue LTP in estrus females, and agonism can induce LTP in diestrus females.

Overall this is a simple, but interesting manuscript that is important for the field.

Response: We are glad that the reviewer appreciates the importance of our results.

There were a few relatively minor comments that should be addressed:

How are the antagonists working in slice. Is there estradiol tone?

Response: Based on studies in rats, there is estradiol tone in the dorsal striatum of both males and females, and the tone correlates with plasma levels of estradiol (Bixo et al., 1986; del Pino et al., 2017). Another source besides blood that supplies estradiol to the striatum is local conversion of testosterone into estradiol via aromatase. A study that used slice electrophysiology to test the effect of estradiol on long-term potentiation (LTP) in the dorsolateral striatum found that not only an estradiol receptor (ER) antagonist but also an aromatase inhibitor affects LTP (Tozzi et al., 2015). Thus, it would be expected that there is a basal level of ER activity occurring in slice, and that antagonists would disrupt this activity in estrus (lines 187 to 189).

How were the doses chosen for the pharmacology studies? The doses are listed but no information on how they were chosen is provided.

Response: We chose the drug doses based on what previous research in slice electrophysiology has used. The Methods section is now updated (lines 134 to 136) with an explanation of how the drug doses were chosen. Thank you for bringing this to our attention.

“Estradiol, the predominant form of the sex hormone estrogen” is stated in the first sentence of the abstract - Estrogen is not a hormone, it is a class of hormones (estrone, E1; estradiol, E2; and estriol, E3). The authors should go through out and make sure that their language is appropriate throughout.

Response: Thank you for this correction. The wording is now fixed (Abstract line 2 and Introduction line 31). We have carefully proof-read the entire manuscript to ensure that our language is accurate.
